# Outcomes Following Balloon Pulmonary Valvuloplasty in Symptomatic Neonates With Tetralogy of Fallot—A CCRC Study

**DOI:** 10.1016/j.jscai.2023.101062

**Published:** 2023-07-05

**Authors:** Shabana Shahanavaz, Athar M. Qureshi, Christopher J. Petit, Bryan H. Goldstein, Andrew C. Glatz, Holly Bauser-Heaton, Courtney E. McCracken, Michael S. Kelleman, George T. Nicholson, Jeffrey D. Zampi, Joelle Pettus, Jeffery Meadows, Kristal M. Hock, Mark A. Law

**Affiliations:** a Division of Pediatric Cardiology, Washington University School of Medicine, St. Louis, Missouri; b Cincinnati Children’s Hospital Medical Center, Cincinnati, Ohio; c Baylor College of Medicine, Texas Children’s Hospital, Houston, Texas; d Emory University School of Medicine, Children’s Healthcare of Atlanta, Atlanta, Georgia; e Columbia University Vagelos College of Physicians and Surgeons, NewYork-Presbyterian Morgan Stanley Children’s Hospital, New York, New York; f UPMC Children’s Hospital of Pittsburgh, Pittsburgh, Pennsylvania; g The Cardiac Center, Children’s Hospital of Philadelphia, Philadelphia, Pennsylvania; h The Perelman School of Medicine, University of Pennsylvania, Philadelphia, Pennsylvania; i Division of Pediatric Cardiology, Ann and Monroe Carell Jr. Children’s Hospital at Vanderbilt, Nashville, Tennessee; j Division of Pediatric Cardiology, University of Michigan, Ann Arbor, Michigan; k Division of Pediatric Cardiology, University of California, San Francisco, California; l Division of Pediatric Cardiology, University of Alabama at Birmingham, Birmingham, Alabama

**Keywords:** balloon valvuloplasty, cyanosis, neonatal, pulmonary valve stenosis, tetralogy of Fallot

## Abstract

**Background:**

Complete repair (CR) can be delayed in neonates with symptomatic tetralogy of Fallot (sTOF) using surgical or transcatheter palliation to relieve cyanosis. Balloon pulmonary valvuloplasty (BPV) is an established treatment for pulmonary valve stenosis; however, its effectiveness in palliating neonates with sTOF has not been well investigated.

**Methods:**

A retrospective chart review between 2005 and 2017 on neonates with sTOF who underwent initial BPV from 9 participating centers of the Congenital Cardiac Research Collaborative was performed. Primary outcome was CR at >30 days after BPV without interval reintervention (RI).

**Results:**

In total, 47 neonates with sTOF underwent BPV, of whom 27 (57%) underwent CR at >30 days after BPV without RI. The median time to CR was 151 days (106-210). RI before CR occurred in 17 patients (36%): surgical shunt (n = 7), outflow tract stenting (n = 6), patent ductus arteriosus stenting (n = 2), and surgical outflow patch (n = 2). Valve-sparing repair at CR was performed in 6 patients (13%) after initial BPV. RI or CR ≤30 days from BPV was associated with smaller infundibular diastolic diameter (*P* = .004). An infundibular diastolic diameter of <3.4 mm demonstrated 75% sensitivity and 67% specificity to predict early CR or RI.

**Conclusions:**

BPV can be an effective palliative therapy in select neonates with sTOF to delay CR. A smaller diastolic infundibulum diameter is a predictor of RI or early CR, and valve-sparing repair is uncommon, making patient selection and alternative palliative methods key when considering BPV palliation in neonates with sTOF.

## Highlights

• Balloon pulmonary valvuloplasty can delay complete repair in symptomatic neonates with tetralogy of Fallot.
• Smaller diastolic infundibulum diameter is an important predictor of reintervention/repair.
• Transannular patch at the time of repair in this population is common; thus, right ventricular outflow tract stenting might more durable.

## Central Illustration

**Figure:**
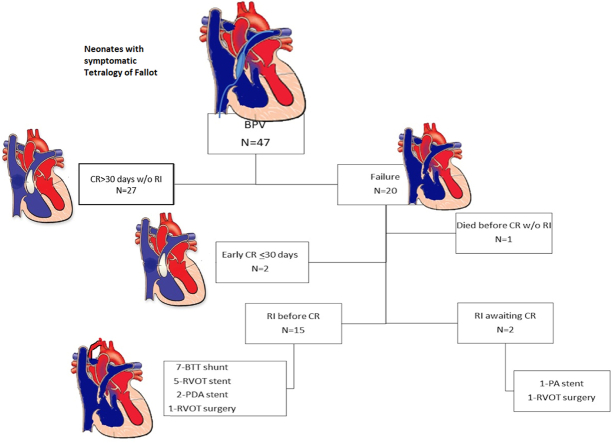

## Introduction

Most of the infants born with tetralogy of Fallot (TOF) are asymptomatic with minimal cyanosis. Neonates with symptomatic tetralogy of Fallot (sTOF) require either a staged repair consisting of an initial palliation before later complete repair (CR) or a primary repair.^1^, ^2^, ^3^ There is significant anatomical variability accounting for the etiology and degree of cyanosis in these patients with sTOF such as hypoplastic infundibulum, pulmonary valve (PV) annulus, and main and branch pulmonary arteries. The variations and combinations influence the possible treatment options. Initial palliation can include a surgical aortopulmonary shunt, right ventricular outflow tract (RVOT) surgery, or transcatheter techniques. Delaying CR may decrease the morbidity and mortality that can be associated with neonatal cardiac surgery.^4^ Surgical shunts have been historically favored but are associated with significant mortality and morbidity.^5^^,^^6^ Stent placement in the ductus arteriosus has been established as a viable alternative to augment pulmonary blood flow but is known to have a higher incidence of reintervention (RI), often to delay surgery to allow for interval somatic growth.^7^, ^8^, ^9^, ^10^ Stent placement in the RVOT has also been used to delay surgery but necessitates the need for transannular patch (TAP) at the time of CR.^7^, ^11^, ^12^

Balloon pulmonary valvuloplasty (BPV) is an established treatment for PV stenosis and has been used to treat sTOF with varying outcomes.^13^, ^14^, ^15^ BPV can be used to augment pulmonary blood flow and allow for interval patient growth before CR.^16^ Some studies have shown that BPV may reduce the need for TAP and allow for the growth of the branch pulmonary arteries before CR.^17^ However, the utility of BPV for sTOF is questionable regarding delay in CR without the need for RI. The primary goal of this study was to evaluate the durability of BPV to palliate sTOF and to identify patient or anatomic features that would favor BPV as an initial palliative approach.

## Methods

We performed a multicenter retrospective cohort study that included all neonates with sTOF who underwent BPV as their initial intervention ≤30 days of age between January 1, 2005, and November 30, 2017, at the 9 centers of the Congenital Cardiac Research Collaborative (CCRC).^17^ Patients were excluded if they showed the following: (1) nonconfluent branch pulmonary arteries; (2) TOF with atrioventricular canal; (3) TOF without PV syndrome; (4) TOF with major aortopulmonary collateral arteries that underwent unifocalization (or there was the intent for unifocalization); or (5) double outlet right ventricle. All medical records were reviewed through January 31, 2019. Previous publications have described the CCRC and the collaborative’s primary results.^2^^,^^18^ The CCRC included (at the time of this study) investigators from the Children’s Hospital of Philadelphia, Cincinnati Children’s Hospital, Children’s Healthcare of Atlanta, UCSF Benioff Children’s Hospital, Monroe Carell Jr. Children’s Hospital at Vanderbilt, Texas Children’s Hospital, St. Louis Children’s Hospital, C.S. Mott Children’s Hospital, and Children’s Hospital of Alabama. Chart abstraction was performed at the center level with deidentified data entered into an electronic database hosted at Children’s Healthcare of Atlanta, which served as the data coordinating center for the CCRC. A remote auditing process was performed to insure quality and accuracy.^19^ This study was approved by the institutional review board at Cincinnati Children’s Hospital, which acted as the single institutional review board of record for each of the participating centers, with a waiver of the need for informed consent.

### Outcomes

The primary objective was to describe outcomes after BPV in this cohort. The primary outcome of this study was successful BPV palliation defined as CR performed >30 days after the BPV without interval RI. RI included all procedures to augment pulmonary blood flow. RIs were defined as surgical—aortopulmonary shunt or RVOT revision (right ventricle to pulmonary artery conduit or RVOT patch) or catheter-based—RVOT stent or ductus arteriosus stent. The 30-day minimum cutoff for success was chosen based on the avoidance of the need for complete surgical correction during the potentially more vulnerable neonatal period. Secondary outcomes included preservation of the PV with avoidance of TAP at the time of CR. BPV procedural and CR outcomes of intensive care unit (ICU) and hospital length of stay, survival, and complications were also evaluated. BPV and CR complications were defined as a complication within 72 hours of the index procedure.

As a secondary objective, we sought to identify factors associated with an unsuccessful BPV palliation. Candidate variables included standard demographic variables of age, weight, preprocedural intubation, preprocedural prostaglandin infusion, echocardiographic and angiographic measures of PV annulus, balloon diameter, angiographic infundibular length and minimum diameter in diastole, and pulmonary artery size.

### Statistical analysis

Statistical analyses were performed using SAS v9.4 (SAS Institute). Statistical significance was assessed at the .05 level of significance. Descriptive statistics are presented as numerical counts with percentages for categorical variables and median (25th-75th percentile) for nonnormally distributed continuous variables. Continuous data were compared between successful palliation and unsuccessful (need of RI or CR ≤30 days) using Wilcoxon rank-sum tests for nonparametric continuous variables. Categorical variable comparison was performed using the χ^2^ or Fisher exact test when counts were <5. Competing risk analysis curves comparing time to early CR or RI, CR, and alive without CR were created. Receiver operator characteristic (ROC) curves were generated to compare the discriminatory performance of PV annulus, infundibular diameter (examined both as an absolute measurement and indexed to patient body surface area) and successful palliation. ROC curves were generated, and sensitivity and specificity estimates were generated using different cutoff values. Cutoff values were derived to maximize accuracy and sensitivity.

## Results

Of the 342 total neonates who underwent initial palliation for sTOF, 47 (14%) underwent BPV as a first intervention. Baseline patient characteristics are demonstrated. Median age at BPV was 7 days (4-14). Thirty (64%) neonates were on prostaglandin therapy, and 14 (40%) were intubated before the procedure. The median balloon-to-annulus ratio was 1.3 (1.2-1.4).

Forty-four neonates (94%) survived through CR with 1 interstage death and 2 patients with RI awaiting CR at the termination of this study (Central Illustration). Twenty-seven patients (57%) underwent a successful CR at >30 days after BPV without interval RI. RI before CR was common (17 [36%]), with aortopulmonary shunt placement being the most frequent RI (7 [41%]); 2 patients required CR earlier than 30 days, and 1 patient died before CR (Central Illustration). Valve-sparing repair was performed in 6 patients in this cohort (5 successful BPV and 1 unsuccessful BPV). TAP was used in 37 patients, with 1 patient receiving a right ventricle–pulmonary artery conduit. No differences in baseline patient characteristics were noted between successful and unsuccessful BPV palliation cohorts except for infundibular diastolic diameter, which was significantly smaller in patients needing early repair or RI (3.7 mm [3.0-5.2] vs 2.4 mm [1.7-3.5]; *P* = .004) (Table 1).

**Central Illustration:**
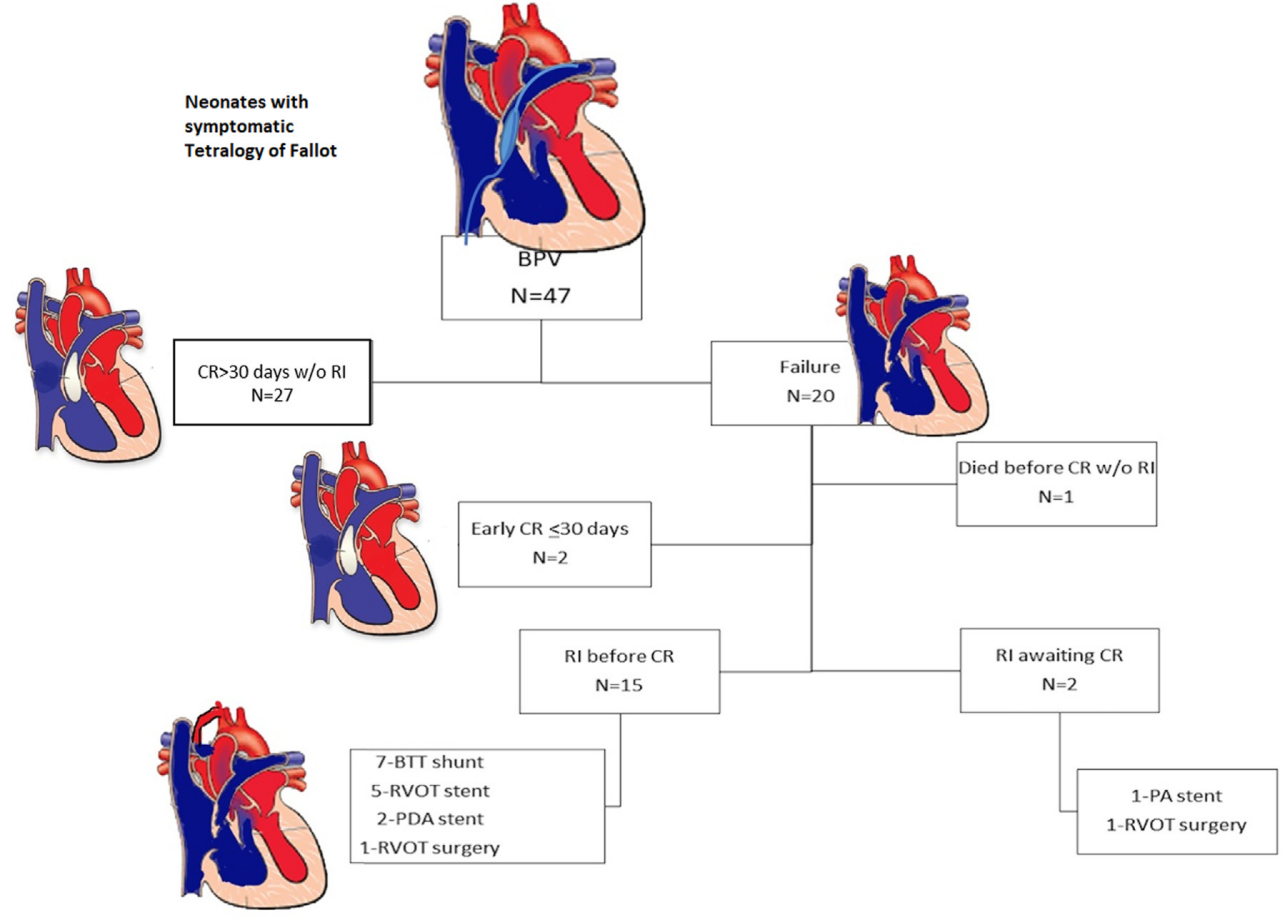
**Flow diagram demonstrating the outcome of neonates who underwent balloon pulmonary valvuloplasty for palliation of symptomatic tetralogy of Fallot.** BPV, balloon pulmonary valvuloplasty; BTT, Blalock–Thomas–Taussig; CR, complete repair; PA, pulmonary artery; PDA, patent ductus arteriousus; RI, reintervention; RVOT, right ventricular outflow tract; w/o, without.

**Table 1:** Baseline characteristics comparing successful balloon pulmonary valvuloplasty (complete repair >30 days without reintervention vs complete repair ≤30 days and/or reintervention prior to complete repair).

Risk factors	CR >30 d without RI (n = 27)	CR ≤30 d and/or RI (n = 20)	*P*
Male sex	13 (48)	14 (70)	.134
Weight at initial intervention, kg	3.1 (2.6-3.5)	2.6 (2.3-3.2)	.088
Prematurity	6 (22)	2 (10%)	.437
Age at initial intervention, d	7 (4-16)	6 (3-10)	.316
Discharged home before BPV	3 (11)	0 (0)	.251
PGE at the time of BPV	15 (56)	15 (75)	.170
Intubated before BPV	8 (30)	6 (30)	.978
PV annulus diameter, mm (n = 46)	4.8 (4.0-5.3)	4.3 (3.5-4.9)	.075
Minimum MPA diameter, mm (n = 36)	4.1 (3.2-4.9)	4.3 (3.3-5.1)	.767
Minimum diastolic infundibular diameter indexed to BSA, mm/m^2^ (n = 41)	18.8 (13.3-23.1)	11.4 (7.4-15.6)	.010
Minimum diastolic infundibular diameter, mm (n = 43)	3.7 (3.0-5.20)	2.4 (1.7-3.5)	.004
Length of infundibulum, mm (n = 42)	7.7 (5.8-9.2)	8.0 (6.8-8.5)	.916
Balloon-annulus ratio (n = 44)	1.3 (1.1-1.3)	1.3 (1.2-1.5)	.435
RPA *z* score (n = 44)	−1.7 (−2.1 to −1.2)	−1.3 (−2.1 to −1.0)	.408
LPA *z* score (n = 44)	−1.5 (−2.1 to −1.0)	−1.48 (−2.1 to −1.1)	.731

Data are presented as n (%) or median (IQR).

BPV, balloon pulmonary valvuloplasty; BSA, body surface area; CR, complete repair; LPA, left pulmonary artery; MPA, main pulmonary artery; PGE, prostaglandin; PV, pulmonary valve; RI, reintervention; RPA, right pulmonary artery.

In Table 2, outcomes between successful BPV palliation versus those who required RI or early repair are compared. Patients who required RI or CR ≤30 days after BPV were more likely to have a longer ICU (*P* = .03) and hospital length of stay (*P* = .025). Eleven (26%) procedural complications occurred, which were more frequent in unsuccessful BPV. There were no BPV procedural deaths, and 1 major adverse event occurred with cardiac tamponade*.* Vascular injury was recorded in 4 patients, 5 patients experienced arrhythmias warranting therapy, and 1 patient presented with neurologic injury. Patients with a successful BPV were less likely to have a complication at the time of CR and shorter hospital and ICU lengths of stay.

**Table 2:** Outcomes comparing successul balloon pulmonary valvuloplasty (complete repair >30 days without reintervention vs complete repair ≤30 days and/or reintervention prior to complete repair).

Outcome	CR >30 d without RI (n = 27)	CR ≤30 d and/or RI (n = 20)	*P*
ICU LOS, d	4 (2-9)	11(4-19)	.030
Total LOS, d	16 (8-26)	26 (16-31)	.025
Complications during BPV	3 (11)	8 (40)	.021
Intubated before CR (n = 43)	0/27 (0)	1/16 (6.3)	.372
Inpatient prior to CR (n = 43)	3/27 (11)	4/16 (25)	.394
Oxygen saturation at CR (n = 42)	89 (83-96)	83 (78-93)	.156
Procedural complications during CR (n = 44)	3/27 (11)	8/17 (47)	.002

Data are presented as n (%) or median (IQR).

BPV, balloon pulmonary valvuloplasty; CR, complete repair; ICU, intensive care unit; LOS, length of stay; RI, reintervention.

**Figure 1:**
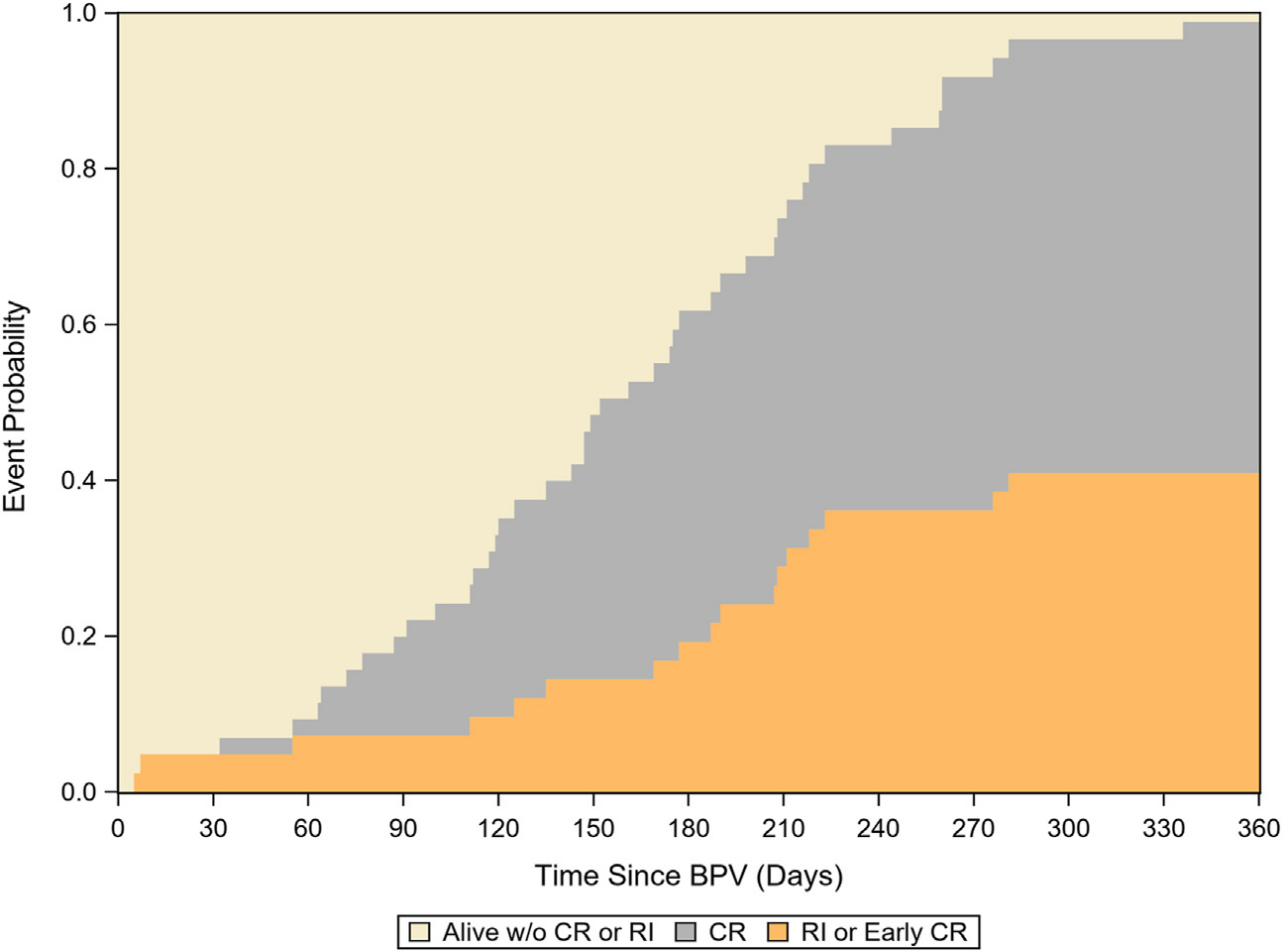
**Competing risk curve demonstrating the various probability of outcomes of CR and reinterventions before CR in patients over time.** BPV, balloon pulmonary valvuloplasty; CR, complete repair; RI, reintervention; w/o, without.

Figure 1 outlines the competing risk analysis of 3 outcomes: (1) alive without CR, (2) CR >30 days after BPV, or (3) early RI or CR ≤30 days after BPV. By 4 months of age, ∼40% of the patients had undergone CR or RI. The median time to CR was 151 days (106-210).

**Figure 2:**
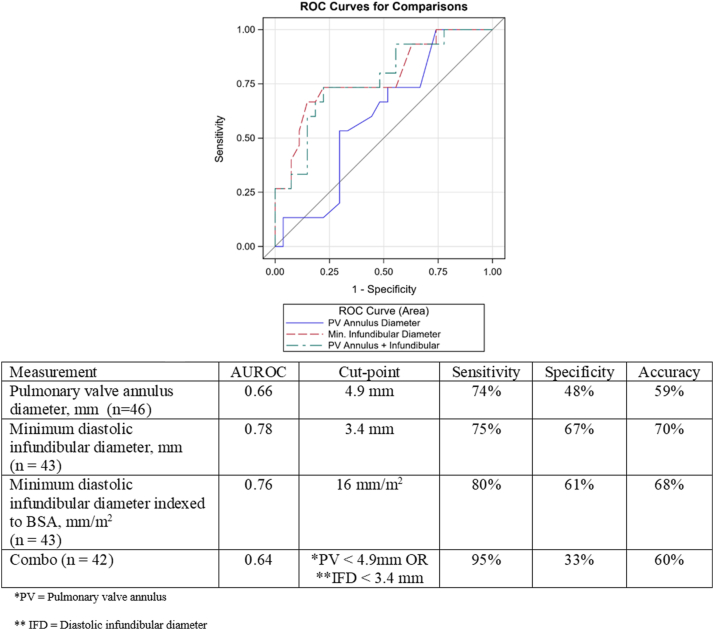
**ROC curve of infundibular diastolic diameter, PV annulus, and infundibular diastolic diameter and pulmonary valve annulus with successful balloon pulmonary valvuloplasty in delay of complete repair >30 days without reintervention.** Min., minimum; PV, pulmonary valve; ROC, receiver operator characteristic.

Using ROC curves to identify the best discriminators of BPV palliation failure demonstrated that the minimal infundibular diameter in diastole as the best predictor of RI than the PV annulus diameter (Figure 2). Optimal cutoffs of infundibular diastolic diameter of <3.4 mm demonstrated a specificity of 67% and sensitivity of 75% for unsuccessful BPV palliation (area under ROC curve, 0.78), and PV annulus diameter of <4.9 mm had a 48% specificity and 74% sensitivity (area under ROC curve, 0.66). Combining both measures did not improve the discriminating ability (area under ROC curve, 0.64). Moreover, indexing the infundibular diameter to body surface area (millimeter per meter squared) did not improve the discriminating ability.

Most of the patients underwent repair using TAP, including those patients with infundibular diameter of ≥3.4 mm (18/20). There was no significant difference in the baseline median PV annulus of patients with valve-sparing repair of 5.0 mm (4.6-5.4) versus TAP of 4.8 mm (4.0-5.5).

## Discussion

In this multicenter study of neonates with sTOF who underwent initial palliative intervention with BPV, we demonstrated that BPV is successful in delaying CR without RI in a subset of sTOF neonates. More than half of the cohort was able to defer CR until older age. Importantly, in ∼40% of the cohort, BPV failed to provide a durable palliation to delay CR >30 days without RI. Furthermore, those patients who recorded an unsuccessful palliation showed evidence of more morbidity such as procedural and CR complications and increased length of stay, suggesting that these failed BPV was associated with a more complicated cardiac course. Most patients ultimately were unable to undergo a PV sparing CR. This study highlights the importance of patient selection to identify those patients in whom the palliative BPV is predictably effective.

We found important anatomic features of the RVOT that are associated with a nondurable BPV palliation. These included measures of RVOT hypoplasia, not only a narrowing of the infundibulum in diastole but also at the level of PV. A minimum diastolic infundibular diameter of <3.4 mm was an important risk factor in this cohort to predict a nondurable BPV palliation. Hypoplasia of the infundibulum has been demonstrated as a predictor of failed BPV in a previous study of older infants; however, the minimum infundibulum predicting RI was larger (24 vs 18 mm/m^2^) by echocardiography than the angiographic measure in this study (19 vs 11 mm/m^2^).^20^ We postulate this difference between the studies is represented by a younger, symptomatic neonatal population and our conservative definition of success in only avoiding CR or RI for ≥30 days. Muneuchi et al^21^ also reported outcomes after BPV in neonates and young infants aged 60 days or younger with PV annulus *z* score of less than −2. In their study, ∼50% of patients after successful BPV required an aortopulmonary shunt within 6 months of BPV with infundibular hypoplasia being a risk factor predicting RI. Their cohort consisted of older patients, of which a significant number did not show symptomatic cyanosis at BPV, which likely affected the finding of later RI.^21^

Patients who are traditionally referred to the cardiac catheterization laboratory for BPV in the setting of sTOF must be carefully evaluated in lieu of other palliative strategies and outcomes. Many patient factors such as gestational age and weight and institution preferences regarding RVOT stenting, Blalock–Thomas–Taussig shunt palliation, and PDA stent placement can play a role. Patients should be preselected based on an echocardiography measurement of less hypoplastic pulmonary annulus and RVOT, including infundibulum. In addition, currently, if a palliative strategy is planned for the neonate with sTOF, consideration of other transcatheter interventions such as RVOT or ductal stent placement should be made as an alternative to BPV, particularly if the infundibular diastolic diameter is <3.4 mm.^10^^,^^22^ RI before CR occurred in 36% of this cohort with the most common RI consisting of aortopulmonary shunts, ductus arteriosus, and RVOT stents, ultimately necessitating 2 significant palliations before achieving a CR. Although we cannot assign causality of failed BPV with increased complications at CR and longer hospital and ICU lengths of stay, there is an important association in these data that could be explained by the increased complexity of care in patients requiring early CR or RIs.

Other studies have demonstrated the growth of PV annulus and branch pulmonary arteries after a staged palliative strategy for sTOF.^1^^,^^14^ Kim et al^17^ evaluated the outcomes of patients who underwent BPV with a similar baseline PV *z* score to the Blalock-Taussig-Thomas shunt group and the infundibulectomy group, but annular growth was superior in the BPV and infundibulectomy group compared with that in the shunt cohort. However, similar pulmonary annular growth after BPV has not been replicated in other studies.^23^^,^^24^

Although we did not serially evaluate annular growth, a surrogate clinically relevant outcome of this measure is the avoidance of TAP at the time of CR. In our cohort of palliated symptomatic cyanotic neonates, only 13% of the patients underwent a valve-sparing CR. The failure of a valve-sparing repair is likely higher given our population of sTOF who warranted intervention within the first 30 days of life. It is very likely that patients who are older might experience a higher likelihood of a valve-sparing repair.^2^ Owing to our relatively small sample size, we were unable to delve further into this subset, but we acknowledge the true annular dimension/*z* score might be only one of the several factors included in the decision, particularly problematic in this multicenter study. Obviously, the “need for TAP” is strongly influenced by institutional surgical preferences and tolerance of residual postoperative stenosis. This important outcome, along with the palliative BPV failure rate, should be considered when weighing various palliative options such as the idea that avoiding an RVOT stent allows for avoidance of TAP type repair. Thus, it would seem prudent that only a small percentage of neonates with sTOF with an acceptable infundibulum and acceptable PV annulus be carefully considered and offered BPV as a palliation. Our data support the notion that for those neonates who are not ideal candidates for BPV, offering other palliative strategies (eg, an RVOT stent) will be more durable and successful in delaying CR. This strategy of abandoning BPV in favor or RVOT stent in patients with hypoplastic infundibulum would result in a TAP in most patients at the time of the CR because most RVOT stents are implanted to span the PV annulus to treat the annular/valvar component as well.

A number of limitations are noted in this study such as the retrospective evaluation and nonrandomization of patients who underwent BPV. Despite a multi-institutional study, the overall sample size remains small. Our definition of success is conservative as one might expect a neonatal palliation to delay the next intervention 3-6 months to allow for a significant interval for patients to grow. We did not collect postinterventional pulmonary insufficiency grading to understand how this might have played a role in the timing of surgery and the decision to perform TAP type repair.

## Conclusion

We demonstrate in this multicenter study of neonates with sTOF that BPV can be an effective palliative therapy in only select neonates with sTOF to delay CR without an interval RI. Smaller angiographic diastolic infundibulum diameter is an important predictor of RI or early CR. TAP at the time of CR in this population is common; therefore, avoidance of RVOT stent placement to preserve the PV function is only beneficial in a very small subset of neonates. Patient selection is key when considering BPV for initial palliation in sTOF.

## References

[1] Kanter K.R., Kogon B.E., Kirshbom P.M., Carlock P.R. (2010). Symptomatic neonatal tetralogy of Fallot: repair or shunt?. Ann Thorac Surg.

[2] Goldstein B.H., Petit C.J., Qureshi A.M. (2021). Comparison of management strategies for neonates with symptomatic tetralogy of Fallot. J Am Coll Cardiol.

[3] Bailey J., Elci O.U., Mascio C.E., Mercer-Rosa L., Goldmuntz E. (2020). Staged versus complete repair in the symptomatic neonate with tetralogy of Fallot. Ann Thorac Surg.

[4] Dorobantu D.M., Mahani A.S., Sharabiani M.T.A. (2018). Primary repair versus surgical and transcatheter palliation in infants with tetralogy of Fallot. Heart.

[5] Al Habib H.F., Jacobs J.P., Mavroudis C. (2010). Contemporary patterns of management of tetralogy of Fallot: data from the Society of Thoracic Surgeons Database. Ann Thorac Surg.

[6] Gladman G., McCrindle B.W., Williams W.G., Freedom R.M., Benson L.N. (1997). The modified Blalock-Taussig shunt: clinical impact and morbidity in Fallot’s tetralogy in the current era. J Thorac Cardiovasc Surg.

[7] Shahanavaz S., Qureshi A.M., Petit C.J. (2021). Factors influencing reintervention following ductal artery stent implantation for ductal-dependent pulmonary blood flow: results from the Congenital Cardiac Research Collaborative. Circ Cardiovasc Interv.

[8] Li D., Zhou X., Li M. (2021). Arterial duct stent versus surgical shunt for patients with duct-dependent pulmonary circulation: a meta-analysis. BMC Cardiovasc Disord.

[9] Glatz A.C., Petit C.J., Goldstein B.H. (2018). Comparison between patent ductus arteriosus stent and modified Blalock-Taussig shunt as palliation for infants with ductal-dependent pulmonary blood flow: insights from the Congenital Catheterization Research Collaborative. Circulation.

[10] Alsagheir A., Koziarz A., Makhdoum A. (2021). Duct stenting versus modified Blalock-Taussig shunt in neonates and infants with duct-dependent pulmonary blood flow: a systematic review and meta-analysis. J Thorac Cardiovasc Surg.

[11] Abumehdi M., Al Nasef M., Mehta C. (2020). Short to medium term outcomes of right ventricular outflow tract stenting as initial palliation for symptomatic infants with complete atrioventricular septal defect with associated tetralogy of Fallot. Catheter Cardiovasc Interv.

[12] Sandoval J.P., Chaturvedi R.R., Benson L. (2016). Right ventricular outflow tract stenting in tetralogy of Fallot infants with risk factors for early primary repair. Circ Cardiovasc Interv.

[13] McCrindle B.W., Kan J.S. (1991). Long-term results after balloon pulmonary valvuloplasty. Circulation.

[14] Lingaswamy D., Koepcke L., Krishna M.R. (2020). Catheter-based palliation for infants with tetralogy of Fallot. Cardiol Young.

[15] Lizano Santamaria R.W., Gillespie M.J., Dori Y., Rome J.J., Glatz A.C. (2015). Palliative balloon pulmonary valvuloplasty for infants with unrestrictive ventricular septal defect or single ventricle associated with severe pulmonary stenosis. Catheter Cardiovasc Interv.

[16] Rao P.S. (2020). Role of palliative balloon pulmonary valvuloplasty in babies with tetralogy of Fallot. Heart Vessels.

[17] Kim G., Ban G.H., Lee H.D., Sung S.C., Kim H., Choi K.H. (2016). Effects of balloon pulmonary valvuloplasty as preoperative palliation for tetralogy of Fallot. Congenit Heart Dis.

[18] Petit C.J., Qureshi A.M., Glatz A.C. (2019). Comprehensive comparative outcomes in children with congenital heart disease: the rationale for the Congenital Catheterization Research Collaborative. Congenit Heart Dis.

[19] Pettus J.A., Pajk A.L., Glatz A.C. (2021). Data quality methods through remote source data verification auditing: results from the Congenital Cardiac Research Collaborative. Cardiol Young.

[20] Rhodes J., O’Brien S., Patel H., Cao Q.L., Banerjee A., Hijazi Z.M. (2000). Palliative balloon pulmonary valvuloplasty in tetralogy of Fallot: echocardiographic predictors of successful outcome. J Invasive Cardiol.

[21] Muneuchi J Watanabe M., Sugitani Y. (2020). Early palliative balloon pulmonary valvuloplasty in neonates and young infants with tetralogy of Fallot. Heart Vessels.

[22] Santoro G., Capozzi G., Caianiello G. (2009). Pulmonary artery growth after palliation of congenital heart disease with duct-dependent pulmonary circulation: arterial duct stenting versus surgical shunt. J Am Coll Cardiol.

[23] Sreeram N., Saleem M., Jackson M. (1991). Results of balloon pulmonary valvuloplasty as a palliative procedure in tetralogy of Fallot. J Am Coll Cardiol.

[24] Heusch A., Tannous A., Krogmann O.N., Bourgeois M. (1999). Balloon valvoplasty in infants with tetralogy of Fallot: effects on oxygen saturation and growth of the pulmonary arteries. Cardiol Young.

